# Temporal Economy

**Published:** 1861-10

**Authors:** 


### Editorial.

### TEMPORAL ECONOMY.

A BROTHER of the *Cosmos* trio doesn't want us to fool away our time; and he thinks we did it in trying to straighten up himself and his brethren of the Pennsylvania Association on the amalgam question. But, then, as we intimated at the outset, we had no hope or intention of converting them. We regarded them as Ephraimites—*joined to their idols*, the said idols being tin, silver and mercury, an unholy trinity of three metals in one cement, the workmanship of their own hands.

We are solemnly told that "no amount of invective or ridicule, emanating from whatever source it may, can influence their views, or deter them from giving utterance to their honest convictions," which is probably all true, especially as it is not likely that either invective or ridicule has been, or will be used by any one for the purpose of interfering with the aforesaid utterance; and it is probably quite as true that no amount of *whining*, "emanating from whatever source it may," will deter us from a kindly criticism of these same utterances, when we think the interests of our profession require it.

The editor copies one of our articles on the "Amalgam Question," (for which we thank him; for it is a pretty good article—perhaps, the best of the series) with the editorial preface that it "is one well worthy of careful perusal; but in presenting it, we can not refrain from indulging an expression of regret that one who is so capable of adding to the knowledge of others, particularly in a field so little cultivated as dental chemistry, should be willing to waste so much valuable time in a direction which neither tends to the advancement of himself or others. This is said with all sincerity and kindness," etc.

Now, this is rather complimentary; and we, too, feel how little the "field of dental chemistry" is cultivated, and, therefore, regret all the more to see those who are "so capable of adding to the knowledge of others" in other fields, bestrewing this one with brush and rubbish, that must be cleared off before it can be successfully cultivated. The most arduous labor of the teacher is, often, to unteach that which has been taught amiss. Those who defile the land should not default the husbandman for expending toil in purifying it which would, otherwise, have been spent in the production of a new crop. We would be glad to have nothing to do but to search for new truths, and we are happy when able to suggest new applications for old ones; but when error is promulgated from high places, we have a duty to perform.

As to the "sincerity and kindness" of our friend, it was not necessary to mention them. We would have given him full credit for both without. We take it for granted that our editorial brethren are *sincere;* and we *know* they are *kind.* All we regret is that our *sincerity* and *kindness*, as genuine, we hope, as our brother's, are mistaken for "invective and ridicule." W.

